# Impact of white matter lesions on associations between prehospital blood pressure and outcomes in spontaneous intracerebral hemorrhage

**DOI:** 10.1177/23969873251343495

**Published:** 2025-06-11

**Authors:** Kristin Tveitan Larsen, Silje Holt Jahr, Maiken Nordahl Selseth, Trine Lied-Herland, Vigdis Hillestad, Hege Ihle-Hansen, Else Charlotte Sandset, Ole Morten Rønning, Espen Saxhaug Kristoffersen

**Affiliations:** 1 Department of Neurology, Akershus University Hospital, Lørenskog, Norway; 2 Department of Geriatric Medicine, Oslo University Hospital, Oslo, Norway; 3 Institute of Clinical Medicine, University of Oslo, Oslo, Norway; 4 Department of Diagnostic Imaging, Akershus University Hospital, Lørenskog, Norway; 5 Department of Acute Medicine, Oslo University Hospital, Ullevaal, Oslo, Norway; 6 Department of Medical Research, Bærum Hospital, Vestre Viken Hospital Trust, Norway; 7 Department of Neurology, Oslo University Hospital, Oslo, Norway; 8 The Norwegian Air Ambulance Foundation, Oslo, Norway; 9 Department of General Practice, Institute of Health and Society, University of Oslo, Oslo, Norway

**Keywords:** Blood pressure, Fazekas, intracerebral hemorrhage

## Abstract

**Introduction:**

There are concerns about the safety of intensive blood pressure (BP) lowering in intracerebral hemorrhage (ICH) patients with white matter lesions (WML). We explored the impact of WML on associations between i) prehospital BP, and ii) BP change, and outcomes in acute, spontaneous ICH.

**Patients and methods:**

This retrospective study included consecutive patients with acute spontaneous ICH, admitted 2011–2020. WML on non-contrast computed tomography were categorized as none-to-mild (0–1) or moderate-to-severe (2–3) on the Fazekas scale. The first systolic BP (SBP) and mean arterial pressure (MAP) from the ambulance and admission, and absolute BP change between these time points, were collected. The outcomes were in-hospital mortality, mortality at 180 days, modified Rankin Scale (mRS) scores at 3 months, and hematoma expansion.

**Results:**

Of 548 patients, 260 (47%) had moderate-to-severe WML. Compared to patients with none-to-mild WML, these patients had a stronger association between higher prehospital MAP and in-hospital mortality (*p* interaction 0.017). WML did not modify associations between prehospital BP and other outcomes. WML modified associations between MAP change and in-hospital mortality (*p* interaction 0.049), MAP change and mRS score 3–6 at 3 months (*p* interaction 0.032), and SBP change and mRS score 3–6 at 3 months (*p* interaction 0.022). These outcomes were poorer with greater BP decrease in patients with moderate-to-severe compared to none-to-mild WML.

**Discussion and conclusion:**

In acute ICH, WML modified the influence of prehospital BP and BP change on clinical outcomes, with a trend toward worse outcomes associated with higher prehospital BP and greater spontaneous BP decrease.

## Graphical abstract

**Graphical abstract:**
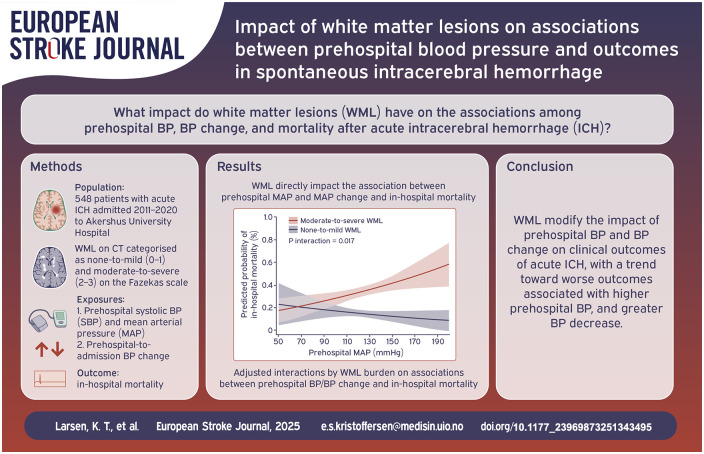

## Introduction

Cerebral white matter lesions (WML) are imaging markers of small vessel disease, the most common cause of spontaneous intracerebral hemorrhage (ICH).^1–3^ These lesions are indicative of damage to the white matter tracts, leading to disrupted communication between different brain regions. WML are a consequence of long-standing hypertension, but also associated with higher age, and other vascular risk factors.^3^ In patients with ICH, presence of cerebral WML is common and associated with mortality and poor functional outcome.^4–6^ However, WML are not clearly associated with hematoma volume or hematoma expansion, which are important outcome predictors in ICH.^6–10^ Thus, the poor outcome associated with WML after ICH may be related to other factors.

In the acute phase of ICH, blood pressure (BP) is often severely elevated, which is associated with hematoma expansion and poor outcome.^11–14^ Current guidelines recommend early, rapid lowering of high BP.^15,16^ Controversy exists as to whether intensive BP lowering in acute ICH promotes cerebral ischemia. The guidelines do not differ for patients with known hypertension or a high burden of WML. Hypothetically, these patients may respond differently to elevated BP or rapid BP changes, because small vessel disease is associated with changed arterial stiffness and pulsatile blood flow.^17–21^ Further, the cerebral autoregulation in the presence of long-standing hypertension may be chronically impaired.^22,23^ Consequently, a rapid BP decrease could more easily lead to cerebral hypoperfusion.

An individual patient data meta-analysis (IPDMA) of the INTERACT2 and ATACH-II trials found that targeting a systolic BP of 120–130 mmHg was safe and associated with less hematoma expansion and better functional outcomes.^24^ A potential U-shaped relationship was observed between the extent of systolic BP reduction in the first hour and functional outcomes, with a 32–46 mmHg reduction being most beneficial, while reductions of ⩾72 mmHg were associated with harm.^25^ Secondary analyses also found that intensive BP reduction was safe regardless of WML burden or baseline BP.^4,9^ However, the trial populations were relatively young, had mostly mild-to-moderate ICH severity, and symptom onset <6 h before treatment, limiting the generalizability of the results for therapeutic decisions in a clinical practice with older and more frail patients.

The aim of the present study was to explore the impact of computed tomography (CT)-assessed WML burden on associations between prehospital BP and outcomes in an unselected population with acute, spontaneous ICH. A secondary aim was to explore the impact of WML burden on associations between spontaneous BP change from the prehospital phase to admission, and outcomes.

## Methods

The population in this retrospective, observational study was drawn from the Akershus Study of Ischemic Stroke and Thrombolysis 1 (ASIST-1), which is a single center cohort that started as a study of ischemic stroke and further developed with inclusion of patients with ICH. The cohort includes consecutively admitted stroke patients at Akershus University Hospital (NCT05378490). The hospital serves both as a primary stroke center and a university hospital. It is Norway’s largest emergency care hospital covering approximately 600.000 inhabitants, about 10% of the Norwegian population. The stroke unit is a comprehensive stroke center, as defined by the European Stroke Organisation.

Patients ⩾18 years old with ICH and admitted within 24 h of symptom onset were included between 1 January 2011 and 31 December 2020. Patients with the following features were excluded: traumatic ICH, ICH after thrombolysis or thrombectomy, hemorrhagic transformation of ischemic stroke, cerebral venous thrombosis, underlying structural vessel malformation or tumor, isolated intraventricular hemorrhage, transfer from another hospital (due to lack of information from the hyperacute, prehospital phase), initial CT performed >24 h after symptom onset, recurrent ICH during the study period (only the first event was included), and missing prehospital data. A flow chart of the study is included as Supplemental Figure S1.

Patient demographics, medical history, and details of the acute event were collected from hospital records. The time from symptom onset or last seen well to admission was categorized as <3, 3–6, 6–12, and 12–24 h. Glasgow Coma Scale (GCS), National Institute of Health Stroke Scale (NIHSS), and modified Rankin Scale (mRS) scores were recorded. If missing, these were retrospectively estimated, if sufficient information was available in the records.^26^ We recorded the first systolic and diastolic BP (SBP and DBP) measured in the emergency medical services (EMS) or referral reports following the patients from the ambulance to admittance, and the first SBP and DBP measured on admission. Mean arterial pressure (MAP) was calculated as (SBP + 2DBP)/3. BP change was the absolute, spontaneous difference between in-hospital and prehospital measurements. There is no active treatment for BP given in the ambulance. All BP were measured as part of routine clinical practice.

Non-contrast CT images were reviewed by study-dedicated neuroradiologists and a radiology resident. The CT images were the first scans performed upon admission for patients with acute stroke symptoms. Hematoma volumes were measured by two radiologists using different methods depending on the location of the hematoma. For non-cerebellar hematomas, the modified ABC/2 method was applied (A = greatest diameter, B = greatest diameter perpendicular to A, C = CT slice count weighted by the area of blood compared to the largest hematoma slice area).^27^ Cerebellar hematomas, expected to be smaller, were measured by manually summing the hematoma areas across all slices and multiplying by slice thickness.^28^ For patients with a follow-up CT performed within 25 h of admission, follow-up hematoma volumes were also estimated. WML in the deep white matter was graded on the Fazekas scale (0–3), where 0 indicated no WML, 1 indicated punctuate foci, 2 indicated beginning confluence, and 3 indicated large confluent areas.^29^ WML burden was defined as none-to-mild for Fazekas scores 0–1 and moderate-to-severe for scores 2–3.

The five outcomes assessed were in-hospital mortality, mortality at 180 days, distribution of mRS scores at 3 months, mRS score 3–6 at 3 months, and hematoma expansion. Hematoma expansion was defined as an increase in volume of > 6 mL and/or >33% from the initial to the follow-up CT.^30,31^ The Norwegian National Population Register provides dates of death for all deceased Norwegian residents, these data are continuously updated in electronic hospital records.

This manuscript follows the STROBE reporting guidelines. Data related to the manuscript is available from the senior author (ESK) upon reasonable request.

## Statistical methods

Continuous variables are reported as medians with interquartile ranges (IQR) and categorical variables as proportions (n/N) with percentages. Statistical significance is defined as *p* < 0.05, using two-sided level of significance. We did not correct for multiplicity, given the exploratory study design. Complete case analyses were performed, excluding patients with missing variables from the multivariable models. STATA statistical software (StataCorp. 2021. *Stata Statistical Software: Release 18*. College Station, TX: StataCorp LLC) was used for the analyses.

Baseline characteristics, prehospital BP, and outcomes were compared between patients with none-to-mild and moderate-to-severe WML, using the Mann-Whitney-U test for the medians and the Pearson’s chi-squared test for the proportions.

In the regression analyses, prehospital SBP and MAP levels were analyzed as continuous per 5 mmHg increment. The prehospital SBP and MAP change were analyzed on a continuous scale, ranging from negative (decrease) to positive (increase) change per 5 mmHg increment. The associations between BP parameters and outcomes were stratified by WML burden and explored in multivariable regression models, adjusted for age, sex, pre-ICH mRS score, preceding antihypertensive, antiplatelet, and anticoagulant treatment, initial hematoma volume, NIHSS score on admission, and time from onset to admission. Models for BP change were additionally adjusted for prehospital BP level. Distribution of mRS scores were analyzed by ordinal logistic regression. The other outcomes were analyzed by binary logistic regression. Non-linear associations between prehospital BP or BP change and outcomes, stratified by WML burden, were explored by adding a quadratic term to the multivariable regression models. Heterogeneity by WML burden was explored by adding an interaction term to the regression models.

## Results

The study included 257 women (47%) and 291 men (53%), aged median 76 (IQR 67–83) years. Fazekas scores were 0 in 149 patients (27%), 1 in 139 patients (25%), 2 in 134 patients (24%), and 3 in 126 patients (23%). Table 1 shows baseline patient characteristics stratified by WML burden. Patients with moderate-to-severe WML were older, more often female, had higher pre-ICH mRS scores, higher NIHSS scores on admission, larger hematoma volumes, and longer time from onset to admission. They more often had a history of ischemic stroke and atrial fibrillation, and use of antithrombotic and antihypertensive drugs.

**Table 1.** Baseline patient characteristics (*N* = 548).

Patients characteristics	None-to-mild WML *N* = 288	Moderate-to-severe WML *N* = 260	*p*
Age (years), median (IQR)	70 (57–79)	80 (74–86)	**<0.001**
Female, *n*/*N* (%)	122/288 (42)	135/260 (52)	**0.025**
Pre-ICH mRS score 3–5, *n*/*N* (%)	48/288 (17)	95/259 (37)	**<0.001**
History of ischemic stroke, *n*/*N* (%)	48/287 (17)	67/260 (26)	**0.010**
Coronary artery disease, *n*/*N* (%)	52/288 (18)	54/259 (21)	0.41
Diabetes, *n*/*N* (%)	42/288 (15)	36/260 (14)	0.81
Atrial fibrillation, *n*/*N* (%)	53/288 (18)	71/259 (27)	**0.012**
On antiplatelet drugs, *n*/*N* (%)	102/286 (36)	114/257 (44)	**0.039**
On anticoagulant drugs, *n*/*N* (%)	58/287 (20)	76/257 (30)	**0.011**
On antihypertensive drugs, *n*/*N* (%)	149/286 (52)	155/256 (61)	**0.048**
Time from symptom onset to admission, *n*/*N* (%)			**0.020**
<3 h	179/288 (62)	128/260 (49)	
3–6 h	40/288 (14)	46/260 (18)	
6–12 h	34/288 (12)	47/260 (18)	
12–24 h	35/288 (12)	39/260 (15)	
NIHSS on admission, median (IQR)	9 (3–16)	12 (5–20)	**0.004**
Acute, in-hospital blood pressure lowering treatment, *n*/*N* (%)	151/288 (52)	124/260 (48)	0.27
Initial hematoma volume (mL), median (IQR)	6.9 (2.8–19.3)	10.1 (2.9–32.7)	**0.031**
Supratentorial hematoma location, *n*/*N* (%)	257/288 (89)	223/260 (86)	0.22

IQR: interquartile range; ICH: intracerebral hemorrhage; mRS: modified Rankin Scale; NIHSS: National Institute of Health Stroke Scale; WML: white matter lesions.

Mann-Whitney-U test used for comparisons of medians, and Pearson’s chi squared test for comparisons of proportions. None-to-mild WML = Fazekas scores 0–1, moderate-to-severe WML = Fazekas scores 2–3. Bold indicates *p*<0.05.

Supplemental Table S1 details the patients who did not have a follow-up CT that enabled assessment of hematoma expansion (*n* = 305, 56%). These patients were older, had higher Fazekas scores, pre-ICH mRS scores, and NIHSS scores, longer time from onset to admission, larger hematoma volumes, and worse clinical outcomes, but lower prehospital SBP, than the population with a follow-up CT.

Table 2 shows the BP variables and outcomes stratified by WML burden. Median prehospital SBP and MAP did not significantly differ between the groups. Patients with moderate-to-severe WML had higher rates of in-hospital and 180 days mortality, and poor functional outcome at 3 months, while no significant differences were observed in hematoma expansion.

**Table 2.** Prehospital BP variables and outcomes (*N* = 548).

BP variables and outcomes	None-to-mild WML *N* = 288	Moderate-to-severe WML *N* = 260	*p*
*Prehospital BP*			
Prehospital SBP (mmHg), median (IQR)	174 (150–192)	180 (160–200)	0.071
Prehospital MAP (mmHg), median (IQR)	123 (110–137)	127 (110–138)	0.33
*Outcomes*			
In-hospital mortality, n/N (%)	50/288 (17)	98/260 (38)	**<0.001**
180 days mortality, n/N (%)	80/288 (28)	142/260 (55)	**<0.001**
mRS score 3–6 at 3 months, n/N (%)	165/274 (60)	218/256 (85)	**<0.001**
Hematoma expansion, n/N (%)	41/146 (28)	22/97 (23)	0.35

BP: blood pressure; SBP: systolic blood pressure; MAP: mean arterial pressure; IQR: interquartile range; mRS: modified Rankin Scale; WML: white matter lesions.

Mann-Whitney-U test used for comparisons of medians and Pearson’s chi squared test for comparisons of proportions. None-to-mild WML = Fazekas scores 0–1, moderate-to-severe WML = Fazekas scores 2–3.

Bold indicates *p*<0.05.

From the prehospital phase to admission, SBP decreased in 238 patients (median change −17 mmHg), increased in 278 patients (median change 17 mmHg), and was unchanged in 32 patients. Similarly, MAP decreased in 295 patients (median change −13 mmHg), increased in 222 patients (median change 10 mmHg), and was unchanged in 24 patients. MAP change could not be calculated in 7 patients due to missing DBP measurements in the prehospital phase or on admission.

**Figure 1.**
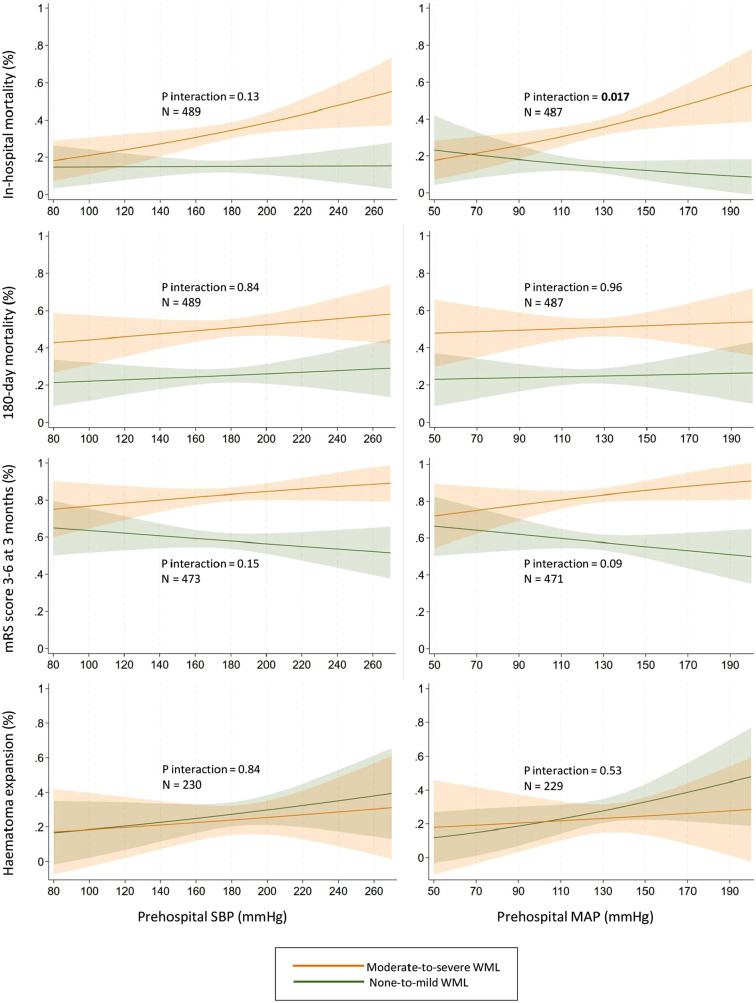
Interactions by white matter lesions burden on associations between prehospital blood pressure and predicted probability of outcomes. Adjusted for age, sex, pre-ICH mRS score, preceding antihypertensive, antiplatelet, and anticoagulant treatment, initial hematoma volume, NIHSS score on admission, and time from onset to admission. None-to-mild WML = Fazekas scores 0–1, moderate-to-severe WML = Fazekas scores 2–3. SBP: systolic blood pressure; MAP: mean arterial pressure; mRS: modified Rankin Scale; WML: white matter lesions.

Table 3 presents the associations between prehospital BP and outcomes, along with interactions by WML burden. WML burden modified the associations between prehospital MAP and in-hospital mortality (*p* for interaction = 0.017). Higher prehospital MAP was associated with increased in-hospital mortality in patients with moderate-to-severe WML (OR 1.14, 95% CI 1.04–1.26), but not in those with none-to-mild WML (OR 0.95, 95% CI 0.82–1.09). Although no other significant interactions between WML burden and prehospital BP on outcomes were identified, there was a trend toward stronger associations between high BP and poor clinical outcomes in patients with moderate-to-severe than none-to-mild WML (Figure 1). This trend was not observed for mortality at 180 days or for hematoma expansion.

**Table 3.** Associations between prehospital BP and outcomes, interactions by WML burden (*N* = 548).

Prehospital BP and outcomes	None-to-mild WML *N* = 288	Moderate-to-severe WML *N* = 260	*p* interaction
*Prehospital SBP per 5 mmHg increment*			
In-hospital mortality (OR, 95% CI)	1.00 (0.91–1.11) *N* = 264	**1.10 (1.02–1.18)** *N* = 225	0.13 *N* = 489
180 days mortality (OR, 95% CI)	1.01 (0.94–1.09) *N* = 264	1.04 (0.97–1.11) *N* = 225	0.84 *N* = 489
Distribution of mRS scores at 3 months (cOR, 95% CI)	0.99 (0.95–1.03) *N* = 251	1.04 (0.99–1.10) *N* = 222	0.25 *N* = 473
mRS score 3–6 at 3 months (OR, 95% CI)	0.96 (0.90–1.03) *N* = 251	1.04 (0.93–1.17) *N* = 222	0.15 *N* = 473
Hematoma expansion (OR, 95% CI)	1.04 (0.97–1.10) *N* = 139	1.03 (0.95–1.13) *N* = 91	0.84 *N* = 230
*Prehospital MAP per 5 mmHg increment*			
In-hospital mortality (OR, 95% CI)	0.95 (0.82–1.09) *N* = 263	**1.14 (1.04–1.26)** *N* = 224	**0.017** *N* = 487
180 days mortality (OR, 95% CI)	1.00 (0.90–1.10) *N* = 263	1.02 (0.93–1.13) *N* = 224	0.96 *N* = 487
Distribution of mRS scores at 3 months (cOR, 95% CI)	0.98 (0.92–1.04) *N* = 250	1.03 (0.95–1.11) *N* = 221	0.62 *N* = 471
mRS score 3–6 at 3 months (OR, 95% CI)	0.94 (0.86–1.04) *N* = 250	1.11 (0.94–1.32) *N* = 221	0.09 *N* = 471
Hematoma expansion (OR, 95% CI)	1.07 (0.98–1.17) *N* = 139	1.02 (0.91–1.15) *N* = 90	0.53 *N* = 229

SBP: systolic blood pressure; MAP: mean arterial pressure; OR: odds ratio; cOR: common odds ratio; CI: confidence interval; mRS: modified Rankin Scale; WML: white matter lesions.

Adjusted for age, sex, pre-ICH mRS score, preceding antihypertensive, antiplatelet, and anticoagulant treatment, initial hematoma volume, NIHSS score on admission, time from onset to admission. None-to-mild WML = Fazekas scores 0–1, moderate-to-severe WML = Fazekas scores 2–3.

Bold indicates *p*<0.05.

**Figure 2.**
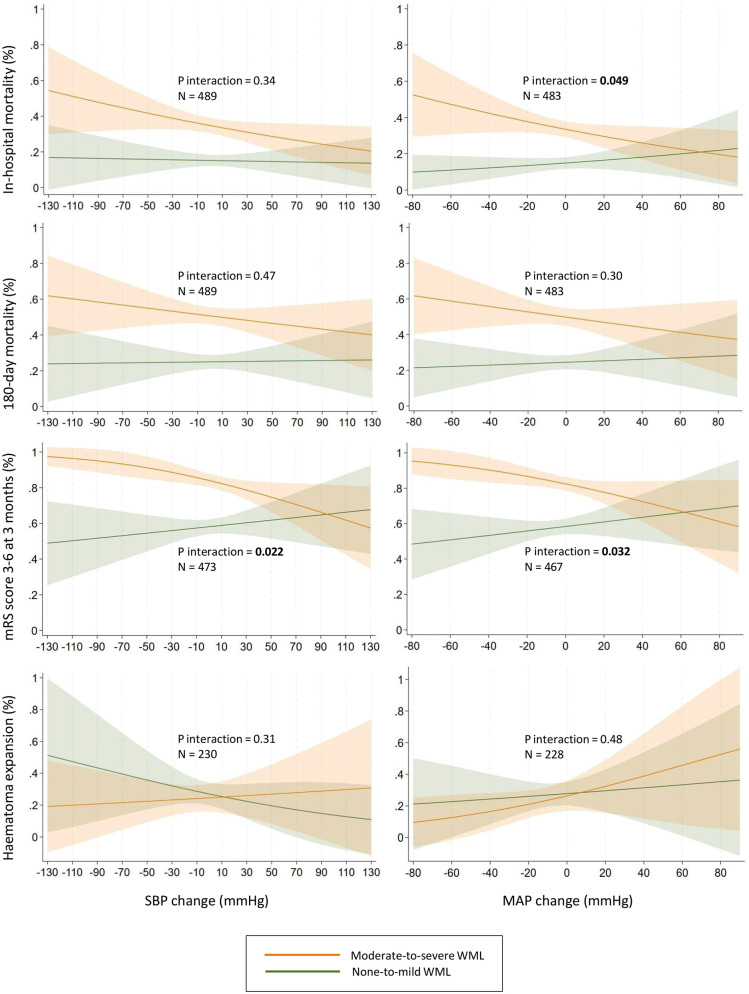
Interactions by white matter lesions burden on associations between blood pressure change from prehospital to admission and predicted probability of outcomes. Adjusted for age, sex, pre-ICH mRS score, preceding antihypertensive, antiplatelet, and anticoagulant treatment, initial hematoma volume, NIHSS score on admission, time from onset to admission, and prehospital blood pressure. None-to-mild WML = Fazekas scores 0–1, moderate-to-severe WML = Fazekas scores 2–3. Negative change = decrease, positive change = increase. SBP: systolic blood pressure; MAP: mean arterial pressure; mRS: modified Rankin Scale; WML: white matter lesions.

Table 4 shows the associations between BP change (ranging from decrease to increase) and outcomes, as well as interactions by WML burden. WML burden modified the association between SBP change and mRS score of 3–6 at 3 months (*p* for interaction = 0.022), between MAP change and in-hospital mortality (*p* for interaction = 0.049), and between MAP change and mRS score of 3–6 at 3 months (*p* for interaction = 0.032). As shown in Figure 2, BP decreases tended to be less favorable for in-hospital mortality and mRS score 3–6 at 3 months in patients with moderate-to-severe than none-to-mild WML. WML burden did not modify the associations between BP change and mortality at 180 days, distribution of mRS scores at 3 months, or hematoma expansion.

**Table 4.** Associations between BP change and outcomes, interactions by WML burden (*N* = 548).

BP change and outcomes	None-to-mild WML *N* = 288	Moderate-to-severe WML *N* = 260	*p* Interaction
*SBP change per 5 mmHg increment*			
In-hospital mortality (OR, 95% CI)	0.96 (0.86–1.06) *N* = 264	0.93 (0.86–1.01) *N* = 225	0.34 *N* = 489
180 days mortality (OR, 95% CI)	1.00 (0.92–1.09) *N* = 264	0.96 (0.89–1.03) *N* = 225	0.47 *N* = 489
Distribution of mRS scores at 3 months (cOR, 95% CI)	1.02 (0.96–1.08) *N* = 251	0.97 (0.92–1.03) *N* = 222	0.12 *N* = 473
mRS score 3–6 at 3 months (OR, 95% CI)	1.02 (0.93–1.12) *N* = 251	0.89 (0.79–1.00) *N* = 222	**0.022** *N* = 473
Hematoma expansion (OR, 95% CI)	0.96 (0.88–1.05) *N* = 139	1.02 (0.94–1.11) *N* = 91	0.31 *N* = 230
*MAP change per 5 mmHg increment*			
In-hospital mortality (OR, 95% CI)	1.00 (0.87–1.15) *N* = 262	0.90 (0.80–1.03) *N* = 221	**0.049** *N* = 483
180 days mortality (OR, 95% CI)	1.02 (0.90–1.15) *N* = 262	0.92 (0.81–1.03) *N* = 221	0.30 *N* = 483
Distribution of mRS scores at 3 months (cOR, 95% CI)	1.02 (0.94–1.11) *N* = 249	0.95 (0.87–1.04) *N* = 218	0.19 *N* = 467
mRS score 3–6 at 3 months (OR, 95% CI)	1.04 (0.91–1.20) *N* = 249	0.85 (0.70–1.03) *N* = 218	**0.032** *N* = 467
Hematoma expansion (OR, 95% CI)	1.02 (0.90–1.16) *N* = 139	1.10 (0.95–1.29) *N* = 89	0.48 *N* = 228

SBP: systolic blood pressure; MAP: mean arterial pressure; OR: odds ratio; cOR: common odds ratio; CI: confidence interval; mRS: modified Rankin Scale.

Adjusted for age, sex, pre-ICH mRS score, preceding antihypertensive, antiplatelet, and anticoagulant treatment, initial hematoma volume, NIHSS score on admission, time from onset to admission, prehospital blood pressure. None-to-mild WML = Fazekas scores 0–1, moderate-to-severe WML = Fazekas scores 2–3.

BP change ranges from negative (decrease) to positive (increase).

Bold indicates *p*<0.05.

There were no significant interactions of WML on non-linear associations between prehospital BP or BP change and outcomes (all *p* for associations and interaction >0.05).

## Discussion

In this retrospective study of 548 patients with spontaneous ICH, WML burden modified the associations between prehospital BP and in-hospital mortality, and between BP change and clinical outcomes. WML burden did not impact associations between prehospital BP parameters and hematoma expansion.

Compared to patients with none-to-mild WML, patients with moderate-to-severe WML had a stronger association between high prehospital MAP and in-hospital mortality. The same tendency was observed for prehospital SBP, and for the other clinical outcomes. High BP in acute ICH is associated with poor outcome^11–14^ and the present findings suggest that patients with a large burden of WML are even more vulnerable to high BP. Current guidelines recommend rapid lowering of high BP in acute ICH,^15,16^ and the present findings support to consider this approach also in patients with a large burden of WML.

On the other hand, the present findings on BP change may warrant extra caution in these patients. The models estimated that BP decrease was more devastating for patients with moderate-to-severe than none-to-mild WML, regarding in-hospital mortality and functional outcome. The BP change in this study was probably relatively rapid due to relatively short transportation distances in the hospital’s catchment area. The findings support the hypothesis that rapid BP decrease could be more harmful in patients with a large WML burden. This could possibly be due to impaired cerebral autoregulation associated with small vessel disease.^17–22^

The WML burden did not modify associations between BP parameters and hematoma expansion. Additionally, no significant differences were observed in the proportions of patients with hematoma expansion between the WML groups. These results are in line with previous studies.^6–10^ However, the analyses of hematoma expansion in the present study are likely hampered by selection bias because a substantial number of patients lacked follow-up CT. Patients with follow-up CT were generally younger, healthier, and had better clinical outcomes. The results regarding hematoma expansion in this study remain uncertain. Therefore, relations between WML, acute BP and hematoma expansion should be investigated further in studies with rigorous imaging protocols.

Previous studies have analyzed WML as continuous, multi-categorical, or dichotomous with different cutoff values, and no gold-standard has been established.^6,9,32,33^ Our dichotomization of Fazekas scores was considered more relevant than a binary presence-or-absence approach, given the expectation of a high prevalence of WML in the study population. This resulted in relatively balanced group sizes. As expected, baseline characteristics differed by WML burden, with patients in the moderate-to-severe WML group being older, having more vascular risk factors, premorbid functional decline, and more severe ICH symptoms. The observed interactions between WML burden and BP parameters in the present study may therefore reflect underlying baseline differences, despite adjustment for relevant confounders.

In line with previous studies, patients with a large WML burden experienced higher mortality and worse functional outcome after ICH.^4–6^ Reasons for the poor outcome observed with WML are still not clear, but may hypothetically be related to dysregulation of cerebral blood flow in the acute phase, impaired neuroplasticity and recovery in the subacute phase, increased risk of subsequent vascular events in the longer term, or other, unknown factors as disability and comorbidities.^3,6,34^

In summary, the present study results suggest that both high acute BP, and rapid BP decrease, are particularly unfavorable in patients with a high WML burden, indicating that acute BP management may be more challenging in these patients. However, the BP decrease in this study was spontaneous and the effects of natural BP reduction may differ from treatment-induced BP decrease. Additionally, a discrepancy was observed between short- and long-term mortality. Early death may have resulted from a rapid BP decrease, or alternatively, frailty in patients who died early may have led to both BP decrease and early death.^35^ Due to the risk of residual confounding, causal inferences cannot be drawn from this study. Based on the nature of the study design it is not possible to draw any firm conclusions about how to treat BP in acute ICH. However, the observed disparities underscore the limited understanding of WML pathophysiology, especially regarding the impact of BP.

The main strength of this study was the large, unselected ICH population with available data on prehospital BP and WML. The use of non-contrast CT images for WML assessment is another strength, as CT is the most widely used first-line imaging modality for acute stroke patients globally, enabling WML evaluation in the hyperacute phase.^29,36,37^ Although magnetic resonance imaging (MRI) is more sensitive and can detect WML at a less advanced stage, CT reliably visualizes WML and may be more specific for identifying clinically relevant cases.^2,29,38,39^ The inclusion of all consecutive ICH patients admitted over a 10-year period to a large, primary hospital reduced selection bias and probably resulted in a reasonably representative ICH population.

Certain limitations should be recognized. Only one BP measurement was recorded from the prehospital phase and on admission. However, using the first measurements minimized the time from symptom onset to measurement, enhancing their relevance to the acute phase of ICH. Not only the difference, but also the speed of BP change between the ambulance and admission measurements may be of interest when investigating the impact of WML on associations between BP and outcomes in ICH. Data on the time interval between the two BP measurements were not available in the present study. There has been shown a beneficial class effect of antihypertensives after initiation of treatment, however, there is still uncertainty about the various agents used to lower BP after acute ICH, and this study cannot provide any further insights into this subject.^40,41^ The ABC/2 method for volume estimation may be less accurate than semiautomated techniques but is widely used in clinical practice and was employed in the present study primarily for estimating hematoma expansion and adjusting regression analyses. The lack of formal frailty score may be regarded as a limitation since frailty is independently associated with early mortality following spontaneous ICH.^35,42^ Chance associations are possible due to the exploratory study design with many outcomes and no adjustment for multiplicity. Nevertheless, the observed findings were fairly consistent and one-directional. Other CT or MRI markers of small vessel disease were not evaluated, such as lacunes or cerebral atrophy, which more thoroughly describe the total burden of small vessel disease.^43^ However, for the acute phase of ICH, identifying one simple parameter from CT that could be used to tailor the acute management may be more useful.

## Conclusion

In acute ICH, patients with a large compared to small WML burden demonstrated a stronger association between high prehospital BP and in-hospital mortality, suggesting that rapid BP lowering may also benefit this group. On the other hand, patients with a large compared to small WML burden had a trend toward worse outcomes with greater BP decrease, highlighting the potential challenges of acute BP management in this population.

Further research that considers underlying brain pathophysiology and the natural course of early BP changes is needed to better establish these associations and interactions, as well as to evaluate the safety and efficacy of rapid blood pressure reduction in ICH patients with a large WML burden. This will help clarify the complex relationship between WML, blood pressure and outcomes.

## Supplemental Material

sj-docx-1-eso-10.1177_23969873251343495 – Supplemental material for Impact of white matter lesions on associations between prehospital blood pressure and outcomes in spontaneous intracerebral hemorrhage Supplemental material, sj-docx-1-eso-10.1177_23969873251343495 for Impact of white matter lesions on associations between prehospital blood pressure and outcomes in spontaneous intracerebral hemorrhage by Kristin Tveitan Larsen, Silje Holt Jahr, Maiken Nordahl Selseth, Trine Lied-Herland, Vigdis Hillestad, Hege Ihle-Hansen, Else Charlotte Sandset, Ole Morten Rønning and Espen Saxhaug Kristoffersen in European Stroke Journal
