# Supplementary material for: Impact of white matter lesions on associations between prehospital blood pressure and outcomes in spontaneous intracerebral hemorrhage
Source: Eur Stroke J. 2025 Jun 11;10(4):1402–12. doi: 10.1177/23969873251343495 (PMC12162525; doi:10.1177/23969873251343495)
Supplement: sj-docx-1-eso-10.1177_23969873251343495 – Supplemental material for Impact of white matter lesions on associations between prehospital blood pressure and outcomes in spontaneous intracerebral hemorrhage [file sj-docx-1-eso-10.1177_23969873251343495.docx]

**Supplemental material**

**Figure S1.** Study selection flow chart


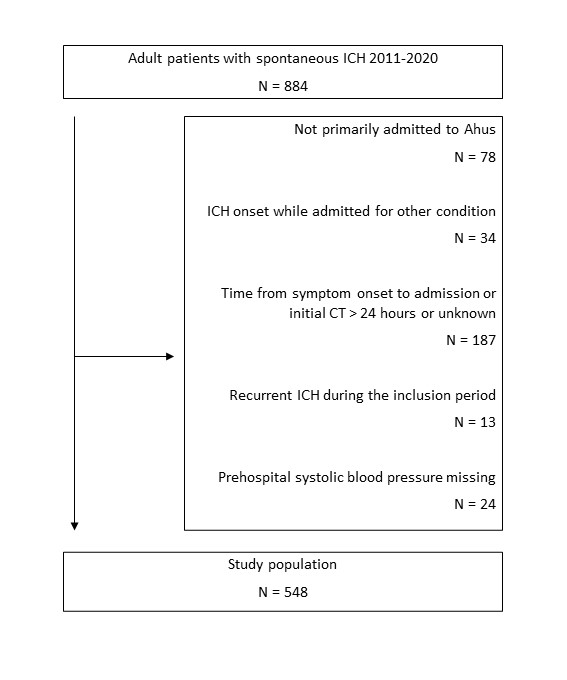


**Table S1.** Patients without and with follow-up CT, available for assessment of hematoma expansion (N=548)

|  | **No follow-up CT**  N=305 | **Follow-up CT**  N=243 | **p** |
| --- | --- | --- | --- |
| Moderate-severe WML, n/N (%) | 163/305 (53) | 97/243 (40) | **0.002** |
| Age (years), median (IQR) | 77 (68-85) | 73 (63-81) | **0.002** |
| Pre-ICH mRS score 3-5, n/N (%) | 102/304 (34) | 41/243 (17) | **<0.001** |
| On antiplatelet drugs, n/N (%) | 120/301 (40) | 96/242 (40) | 0.96 |
| On anticoagulant drugs, n/N (%) | 71/302 (24) | 63/242 (26) | 0.50 |
| *Time from symptom onset to admission, n/N (%)* |  |  | **0.025** |
| *<3 hours* | 157/305 (51) | 150/243 (62) |  |
| *3-6 hours* | 46/305 (15) | 40/243 (16) |  |
| *6-12 hours* | 52/305 (17) | 29/243 (12) |  |
| *12-24 hours* | 50/305 (16) | 24/243 (10) |  |
| NIHSS on admission, median (IQR) | 14 (5-27) | 8 (3-14) | **<0.001** |
| Initial hematoma volume (mL), median (IQR) | 11.9 (3.6-41.6) | 5.9 (2.1-15.5) | **<0.001** |
| In-hospital mortality, n/N (%) | 111/305 (36) | 37/243 (15) | **<0.001** |
| 180 days mortality, n/N (%) | 160/305 (52) | 62/243 (26) | **<0.001** |
| mRS score 3-6 at 3 months, n/N (%) | 237/295 (80) | 146/235 (62) | **<0.001** |
| Prehospital SBP (mmHg), median (IQR) | 175 (150-194) | 180 (160-200) | **0.022** |

Mann-Whitney-U test used for comparisons of medians and Pearson’s chi squared test for comparisons of proportions. Moderate-to-severe WML=Fazekas scores 2-3. *WML, white matter lesions.* *CT, computed tomography. IQR, interquartile range. ICH, intracerebral hemorrhage. mRS, modified Rankin Scale. NIHSS, National Institute of Health Stroke Scale. SBP, systolic blood pressure.*
